# Case report: Successful treatment of a patient with relapsed/refractory primary central nervous system lymphoma with thiotepa-based induction, autologous stem cell transplantation and maintenance

**DOI:** 10.3389/fonc.2023.1333761

**Published:** 2024-01-29

**Authors:** Luyao Wang, Yili Fan, Boxiao Chen, Jiawei Zhang, Luyu Yang, Xi Qiu, Huawei Jiang, Jinfan Li, Xibin Xiao, Liansheng Huang, Yang Xu

**Affiliations:** ^1^ Department of Hematology, The Second Affiliated Hospital, Zhejiang University School of Medicine, Hangzhou, Zhejiang, China; ^2^ Department of Pathology, The Second Affiliated Hospital, Zhejiang University School of Medicine, Hangzhou, China

**Keywords:** primary central nervous system lymphoma, thiotepa, autologous stem cell transplantation, anti-PD-1 antibody, maintenance therapy

## Abstract

Despite significant improvements in prognosis, a subset of patients with primary central nervous system lymphoma (PCNSL) remains at high risk for relapse. The treatment of relapsed and refractory (R/R) PCNSL remains a major clinical challenge. Herein, we present a 24-year-old patient with PCNSL who relapsed 4 years after initial diagnosis and subsequently became refractory to high-dose methotrexate (HD-MTX), temozolomide, whole brain radiation therapy (WBRT), ibrutinib, and lenalidomide. She received thiotepa with anti-programmed cell death protein 1 (PD-1) antibody and achieved partial remission and then underwent autologous stem cell transplantation (ASCT) with thiotepa-based conditioning. Post-transplant maintenance with thiotepa and anti-PD-1 at 3-month intervals resulted in a durable complete response (CR) in this case of R/R PCNSL. Our report highlights the important role of thiotepa in the treatment of patients with R/R PCNSL.

## 1 Introduction

Primary central nervous system lymphoma (PCNSL) is a rare form of non-Hodgkin lymphoma, with diffuse large B-cell lymphoma accounting for 95% of histologies (1, 2). While HD-MTX-based chemotherapy has improved treatment responses, there is a high rate of relapse and a significant number of patients who are resistant to HD-MTX (3). The prognosis for relapsed or refractory (R/R) PCNSL is poor, with a median overall survival of only 2-3 months (4). Novel treatment options for R/R PCNSL include anti-PD-1 antibodies, Bruton tyrosine kinase inhibitors (BTKi), immunomodulatory drugs (IMIDs), and chimeric antigen receptor T (CAR-T) cell therapy, resulting in impressive clinical responses (3, 5). We present a case of R/R PCNSL that was successfully treated by thiotepa-based induction, autologous stem cell transplantation (ASCT) and post-transplant maintenance, and highlight the important role of thiotepa in an era of targeted immunotherapy.

## 2 Case presentation

**Figure 1:**
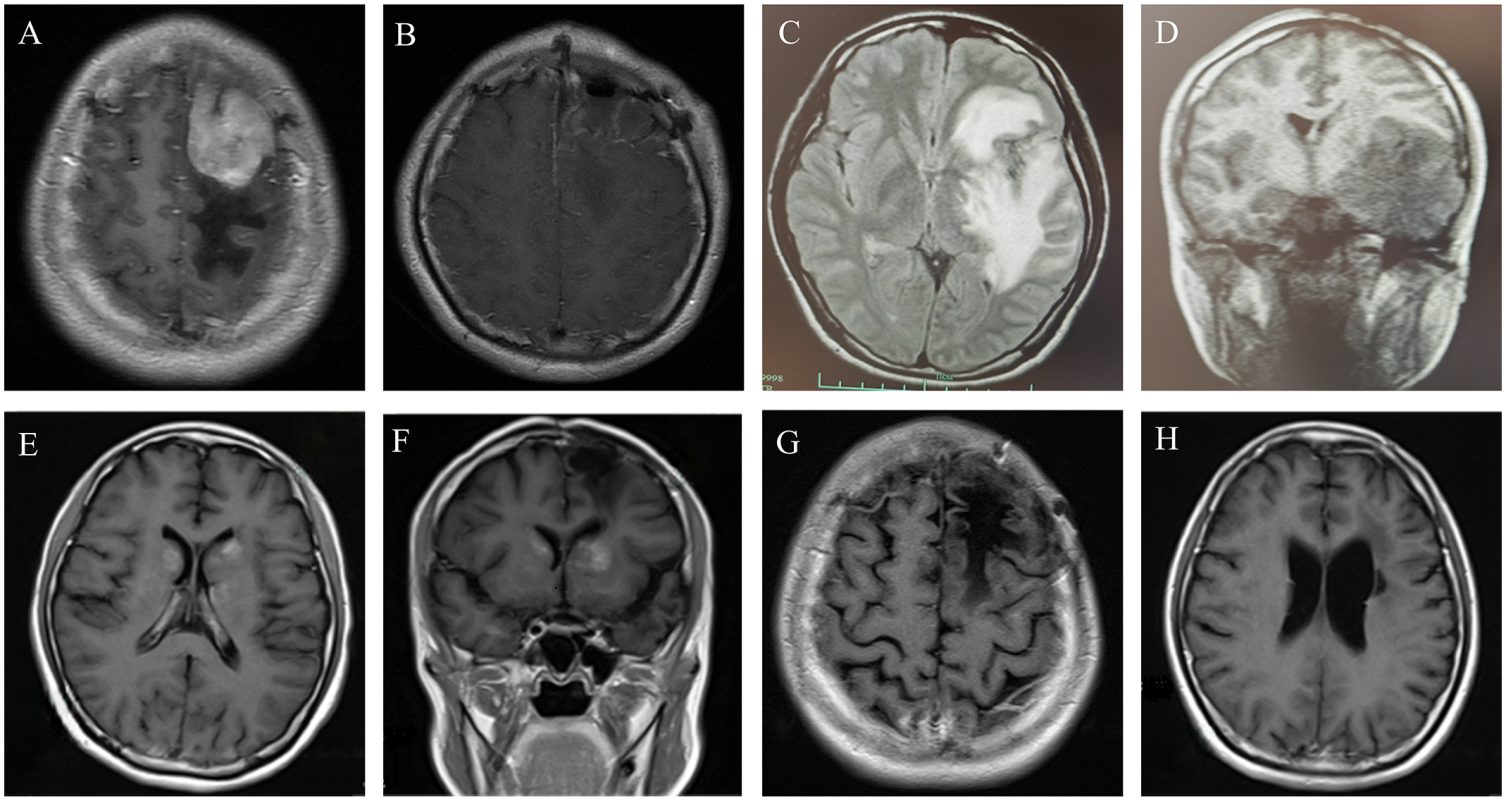
**(A)** Contrast-enhanced MRI revealed a 36*41*37mm homogenously enhancing lesion in the left frontal parietal lobe, with obvious space-occupying effect and peripheral edema. **(B)** Contrast-enhanced brain MRI after left frontal lobe tumor resection. **(C, D)** In September, 2019, the brain MRI showed the mass in the left frontal temporal lobe had clearly enlarged, considering relapse. **(E, F)** Axial T1 weighted image with gadolinium enhancement in February 2021, showing the patch−shaped enhanced masses in the bilateral basal ganglia. **(G, H)** The latest contrast-enhanced brain MRI showed that the masses in the bilateral basal ganglia disappeared.

**Figure 2:**
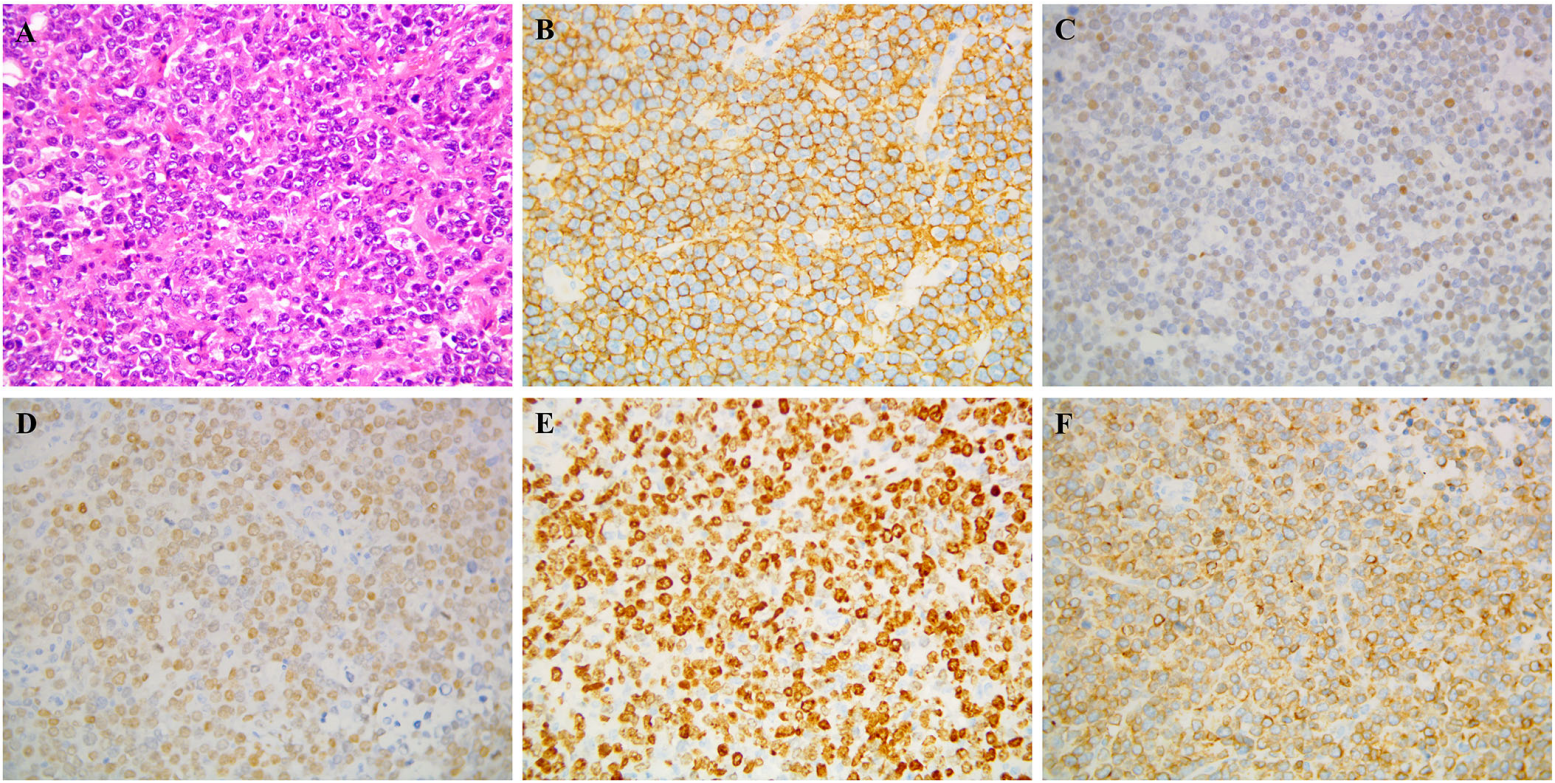
Pathological biopsy: Postoperative specimen showing diffuse neoplastic lymphoid cells on hematoxylin and eosin staining **(A)** Immunocytochemistry demonstrated that the neoplastic lymphocytes were positive for **(B)** CD 20, **(C)** BCL-6, **(D)** MUM-1, **(E)** Ki-67, **(F)** CD79a.

In September 2014, a 24-year-old woman was admitted because of headache, nausea, and vomiting for two weeks. A contrast-enhanced MRI of the brain revealed a hyperintensive lesion located in the left frontal region (**Figure 1A**). Laboratory tests for HIV, HBV, EBV, and CMV were negative. The patient underwent intracerebral tumor resection (**Figure 1B**), and immunohistochemical results indicated that the tumor cells were positive for CD20, CD79a, BCL-6 and MUM-1, and negative for CD3, CD43, BCL-2, CD10, CD30, and ALK, consistent with diffuse large B-cell lymphoma, non-germinal center B-cell (non-GCB) type, with a Ki-67 proliferation index of >90% (**Figure 2**). She was then referred to hematology for further evaluation. A lumbar puncture was performed, and the cerebrospinal fluid (CSF) pressure and results of cerebrospinal fluid analysis were normal. The patient did not undergo slit-lamp examination due to the absence of ocular symptoms. The bone marrow biopsy showed no evidence of lymphoma involvement, and the positron emission tomography/computed tomography (PET/CT) did not detect any lymphoma lesions outside the central nervous system. The patient received six cycles of HD-MTX-based chemotherapy, which included MTX at 3 g/m^2^ on day 1, cytarabine (Ara-C) at 2 g/m^2^ every 12 hours on day 2, and 200mg temozolomide on days 1-5. After completion of chemotherapy in April 2015, brain MRI and PET-CT scans showed a significant reduction in lesion size without any enhanced signals. The CSF analysis results were normal. The patient and her family declined to proceed with ASCT due to concerns over toxicities. She underwent regular follow-up and had remained in complete remission for more than four years until September 2019.

**Figure 3:**
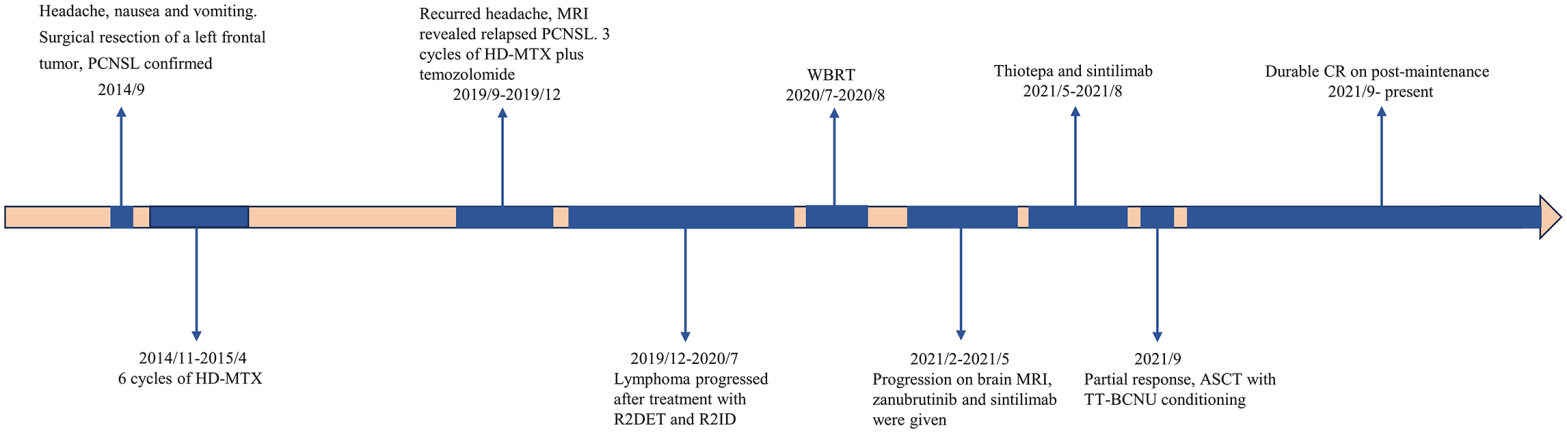
The timeline of symptoms, imaging changes, and clinical interventions since diagnosis.

The patient underwent a brain MRI due to recurrent headaches, which revealed a newly enlarged lesion in the left frontotemporal lobe (**Figures 1C, D**), indicating a relapse of PCNSL. She received three cycles of HD-MTX (3 g/m^2^ on day 1) and temozolomide (220mg on day 1-5), followed by two cycles of R2DET regimen, which consisted of rituximab (RTX) at 600 mg on day 0, lenalidomide 25mg on days 1-21, dexamethasone (DXM) 20 mg on days 1-5, etoposide 100 mg on days 1-5 and temozolomide 300mg on days 1-5. A subsequent MRI revealed new lesions in the right paraventricular basal ganglia, necessitating a switch to the R2ID regimen administered as follows: RTX 600mg on day 0, lenalidomide 25mg on days 1-21, ibrutinib 140mg on days 1-21, and DXM 20mg on days 1-5. However, the tumor continued to progress, and the patient was unable to tolerate intensive chemotherapy, we opted for whole brain radiotherapy (WBRT) in July 2020. After a short period of resolution, a brain MRI in February 2021 revealed significant masses in bilateral basal ganglia (**Figures 1E, F**). Based on the efficacies of BTKi and anti-PD 1 antibody shown in several pilot studies (6, 7), we initiated a treatment plan that includes zanubrutinib 160 mg twice daily on days 1-21 and sintilimab 200 mg on day 1 for 21 days per cycle. The lesions on MRI did not improve after three cycles, and we modified the treatment to sintilimab 200mg on day 1 and thiotepa 40mg/m^2^ on day 3, resulting in a partial remission (PR) as evidenced by the brain MRI. In September 2021, the patient underwent ASCT conditioning with thiotepa and carmustine (TT-BCNU). A post-ASCT MRI revealed complete remission (CR), and maintenance was considered to prevent relapse. Due to a temporary shortage of sintilimab, we administered another anti-PD-1 antibody, tislelizumab 200 mg on day 1 along with thiotepa 40 mg/m^2^ on day 2, at an interval of 3 months for 2 years after transplant. The patient has remained in CR without any evidence of lymphoma, which has been closely monitored (**Figures 1G, H**). The timeline of symptoms, imaging changes, and clinical interventions is summarized in **Figure 3**.

## 3 Discussion

Although HD-MTX-based chemotherapy has significantly improved the prognosis of PCNSL, a significant proportion of patients do not respond to induction therapy, and many relapse after initial response (8). The prognosis of patients with R/R PCNSL is poor, with a median OS of 2-3 months. For patients who have achieved a durable response after prior MTX-based therapy, HD-MTX re-treatment is considered appropriate, with an objective response rate (ORR) of 85% and a CR rate of 75% (9). Furthermore, a recent study demonstrated that the efficacy of HD-MTX was affected by the duration of the first CR and whether whole brain radiotherapy (WBRT) was included in the initial treatment (10). With advances in understanding the pathogenesis of PCNSL, various new small molecules targeting aberrant pathways are increasingly used to treat R/R PCNSL. These include ibrutinib and lenalidomide, which have been recommended by the NCCN guidelines (11). In spite of an impressive response rate of 50-60%, their responses usually last less than 6 months (5). In a phase I study, ibrutinib demonstrated an ORR of approximately 70% in R/R PCNSL (12). However, a phase II study with ibrutinib alone only showed an ORR of 52% and a median PFS of 3.3 months in R/R PCNSL (13). The TEDDi-R treatment, which includes temozolomide, etoposide, liposomal doxorubicin, DXM, ibrutinib, and RTX, achieved an 86% CR rate in patients with R/R PCNSL (14). In addition, the second-generation BTKis with highly selective binding capacity and fewer off-target effects, have been explored for the treatment of R/R PCNSL. For instance, zanubrutinib has shown clinical efficacy when used in combination with chemotherapy or cell therapy (15, 16). Tirabrutinib, an irreversible BTKi approved in Japan (17), resulted in an ORR of 63.6% and a median PFS of 2.9 months in a phase I/II study (18). Currently, several ongoing clinical trials are investigating the efficacy of zanubrutinib alone (NCT05117814) or in combination with pemetrexed (NCT05681195), chemotherapy (NCT05896007), or lenalidomide (NCT04938297) in patients with PCNSL.

The anti-PD-1 monoclonal antibody targets the PD-1/PD-L1 pathway to prevent immune escape and exert anti-tumor activity (19). A case series of four R/R PCNSL patients demonstrated that treatment with nivolumab resulted in over 13 months of PFS for each patient (20). In a prospective phase II study, 13 R/R PCNSL patients who received orelabrutinib and sintilimab had an overall response rate of 61.5%, with a complete response rate of 31% (4/13) (21). In addition, ongoing clinical trials are evaluating the combination of BTK inhibitors with immune checkpoint inhibitors in PCNSL. These trials include NCT04462328 (Phase I, combining acalabrutinib with durvalumab) and NCT03770416 (Phase II, combining ibrutinib with nivolumab). However, the efficacy of BTKi alone or in combination with PD-1 inhibitor versus conventional chemotherapy needs to be evaluated in prospective randomized clinical trials.

Thiotepa, an alkylating agent, is preferred for CNS-based tumors because of its ability to penetrate the blood-brain barrier (BBB) and its superior bioavailability in the CNS (22). The MATRix regimen, consisting of methotrexate, cytarabine, thiotepa, and rituximab, was associated with a significantly improved CR rate of 49% in the randomized phase II IELSG32 trial, compared with a CR rate of 23% in those treated with HD-MTX and Ara-C and 30% in those treated with MTX, Ara-C, plus RTX (23). Furthermore, the long-term follow-up results of the IELSG32 trial confirmed the superiority of the MATRix regimen, with a remarkable 7-year overall survival (OS) rate of 56% compared to 21% in the methotrexate-cytarabine group (24). BCNU/TT, Busulfan/TT, and TBC are commonly used conditioning regimens for HDC-ASCT in PCNSL patients and have demonstrated excellent efficacy. In this case, thiotepa was combined with temozolomide and PD-1 antibody after failing multiple lines of targeted therapy, resulting in an improved response. For consolidation in patients with PCNSL, ASCT and WBRT are feasible options (1). The patient initially received WBRT, which produced a partial response. ASCT is the preferred choice for young patients without prior transplantation (25). Although the strategy and duration of maintenance therapy followed by ASCT remain controversial, the patient received thiotepa and anti-PD-1 every three months for two years and has remained in continuous complete remission.

## 4 Conclusion

R/R PCNSL is common and presents a therapeutic challenge in clinical practice. Novel small molecules that target the B cell receptor or immune evasion have shown promising efficacy, but their use is often limited by short duration. Thiotepa, with its unique high CNS bioavailability, presents new therapeutic opportunities for PCNSL. This case suggested that thiotepa-containing induction and maintenance therapy is a viable option for heavily treated patients with R/R PCNSL.

## Data availability statement

The original contributions presented in the study are included in the article/supplementary material. Further inquiries can be directed to the corresponding author.

## Ethics statement

The studies involving humans were approved by Ethics Committee of the Second Affiliated Hospital of Zhejiang University School of Medicine (Ethics Approval No.2020145). The studies were conducted in accordance with the local legislation and institutional requirements. Written informed consent for participation was not required from the participants or the participants’ legal guardians/next of kin in accordance with the national legislation and institutional requirements. Written informed consent was obtained from the individual(s) for the publication of any potentially identifiable images or data included in this article.

## Author contributions

LW: Writing – original draft, Data curation, Writing – review & editing, Formal analysis. YF: Methodology, Writing – review & editing. BC: Methodology, Writing – review & editing. JZ: Methodology, Writing – review & editing. LY: Methodology, Writing – review & editing. XQ: Resources, Writing – review & editing. HJ: Investigation, Writing – review & editing. JL: Visualization, Writing – review & editing. XX: Validation, Writing – review & editing. LH: Formal analysis, Writing – review & editing. YX: Writing – review & editing, Investigation, Conceptualization, Data curation, Funding acquisition, Writing – original draft.
